# Commentary: The Barlow valve: Understanding disease and symmetry

**DOI:** 10.1016/j.xjtc.2021.10.006

**Published:** 2021-10-09

**Authors:** Carlos A. Mestres, Miguel A. Piñón, Eduard Quintana

**Affiliations:** a Department of Cardiac Surgery, University Hospital Zürich, Zürich, Switzerland; b Department of Cardiothoracic Surgery, The University of the Free State, Bloemfontein, South Africa; c Department of Cardiac Surgery, Hospital Universitario “Alvaro Cunqueiro”, Vigo, Spain; d Department of Cardiovascular Surgery, Hospital Clinic de Barcelona, University of Barcelona, Barcelona, Spain

**Figure:**
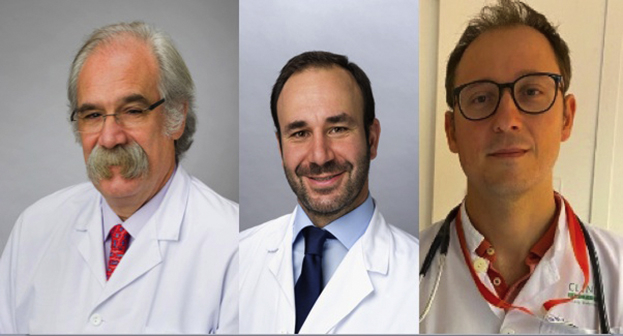
Carlos A. Mestres, MD, PhD, FETCS (*left*), Miguel A. Piñón, MD, PhD (*center*), and Eduard Quintana, MD, PhD, FETCS (*right*)

**Central Message**

This historical summary of the main aspects of the Barlow valve helps readers to understand the complexity of this pathoanatomical and functional disease and the best approach for successful surgery.

See Article page 58.

Mitral valve repair is the gold standard in the surgery of degenerative mitral valve disease, a solidly established option supported by practice guidelines^1^ despite discussion.^2^ The main goal of repair is achieving valve competence.^3^ Of special interest is the complex described by the late John Barlow in 1963, known as the Barlow valve or Barlow disease. His description of late systolic murmur and malignant cardiac arrhythmias triggered by mitral valve prolapse^4^ opened a new era in the investigation of the mitral valve.

The Barlow valve still poses technical challenges to surgeons due to its multifaceted pathoanatomical presentation with bileaflet involvement with abnormally thickened leaflet tissue, gross involvement of the subvalvular apparatus, and annular dilatation.^5^ The main aspects of the disease are too much leaflet tissue and abnormal annular function. Pooling this all together, one understands that correction of the Barlow valve is more complex than for other disease forms. This complexity influences outcomes, although experienced centers report freedom from reoperation for regurgitation of 85% to 90% at 10 years.^6^, ^7^, ^8^

In this issue of the *Journal*, Barlow and colleagues^9^ briefly review this complex anatomical–functional interaction,^9^ a very useful historical summary. As we previously pointed out, history helps in understanding.^10^ This is the case here. Why? John Barlow started from the clinical observation and examination of patients with a mid-systolic click and late systolic murmur and palpitations, based on his interest in cardiac sounds,^11^ before echocardiography. From there, the Barlow valve has been established as individual entity.^5^ Modern echocardiography has helped to better understand the sequence of events until mitral regurgitation becomes significant.^12^^,^^13^ Surgical experiences with follow-up outcomes allowed us to confirm these valves are amenable for repair.^3^

Additional investigations showed that pathoanatomic changes in the form of annular abnormalities like disjunction should not be separated from the disease complex.^14^ The annular “instability” also may play a role in the development of ventricular arrhythmias.^15^ There is ongoing research regarding genetic etiology in mitral valve prolapse, something unknown with few genes identified. Van Wijngaarden and colleagues^16^ suggest some association between cardiomyopathy genes and prolapse, needing further investigation.

Surgery of the Barlow valve aims at restoring competence, releasing leaflet tension, and achieving leaflet coaptation. Another issue is like in other repairs what may eventually happen with the left ventricle^17^ or if surgery should be performed before significant regurgitation develops.^9^ Considering the Barlow valve, symmetry is also an issue, as there is bileaflet billowing and prolapse. The disease cannot easily be replicated as a regular P2 prolapse in a model for bench simulation. Imbrie-Moore and colleagues^18^ developed a cross-species model of the disease for biomechanical analyses of repair techniques in an ex vivo model. With all the limitations in mind, it may help improving knowledge and test the ability to repair.

This elegant and comprehensive historical review of Barlow and colleagues on the Barlow valve^8^ is another call for attention to the importance of reviewing history to understand the present and imagine the future.
